# Infrastructure for the life sciences: design and implementation of the UniProt website

**DOI:** 10.1186/1471-2105-10-136

**Published:** 2009-05-08

**Authors:** Eric Jain, Amos Bairoch, Severine Duvaud, Isabelle Phan, Nicole Redaschi, Baris E Suzek, Maria J Martin, Peter McGarvey, Elisabeth Gasteiger

**Affiliations:** 1 Swiss-Prot Group, Swiss Institute of Bioinformatics, CMU, 1 Michel Servet, 1211 Geneva 4, Switzerland; 2 Department of Structural Biology and Bioinformatics, Faculty of Medicine, University of Geneva, 1 Michel Servet, 1211 Geneva 4, Switzerland; 3 The EMBL Outstation – European Bioinformatics Institute, Wellcome Trust Genome Campus, Hinxton, Cambridge, CB10 1SD, UK; 4 Protein Information Resource (PIR), Georgetown University Medical Center, 3300 Whitehaven Street NW, Washington, DC 20007, USA

## Abstract

**Background:**

The UniProt consortium was formed in 2002 by groups from the Swiss Institute of Bioinformatics (SIB), the European Bioinformatics Institute (EBI) and the Protein Information Resource (PIR) at Georgetown University, and soon afterwards the website  was set up as a central entry point to UniProt resources. Requests to this address were redirected to one of the three organisations' websites. While these sites shared a set of static pages with general information about UniProt, their pages for searching and viewing data were different. To provide users with a consistent view and to cut the cost of maintaining three separate sites, the consortium decided to develop a common website for UniProt. Following several years of intense development and a year of public beta testing, the  domain was switched to the newly developed site described in this paper in July 2008.

**Description:**

The UniProt consortium is the main provider of protein sequence and annotation data for much of the life sciences community. The  website is the primary access point to this data and to documentation and basic tools for the data. These tools include full text and field-based text search, similarity search, multiple sequence alignment, batch retrieval and database identifier mapping. This paper discusses the design and implementation of the new website, which was released in July 2008, and shows how it improves data access for users with different levels of experience, as well as to machines for programmatic access.

is open for both academic and commercial use. The site was built with open source tools and libraries. Feedback is very welcome and should be sent to help@uniprot.org.

**Conclusion:**

The new UniProt website makes accessing and understanding UniProt easier than ever. The two main lessons learned are that getting the basics right for such a data provider website has huge benefits, but is not trivial and easy to underestimate, and that there is no substitute for using empirical data throughout the development process to decide on what is and what is not working for your users.

## Background

The UniProt consortium[1] was formed in 2002 by groups from the Swiss Institute of Bioinformatics (SIB), the European Bioinformatics Institute (EBI) and the Protein Information Resource (PIR) at Georgetown University, and soon afterwards the website  was set up as a central entry point to UniProt resources. Requests to this address were redirected to one of the three organisations' websites (,  and ). While these sites shared a set of static pages with general information about UniProt, their pages for searching and viewing data were different: The SIB was redirecting such requests to the ExPASy website, where some of the data and tools had been available since 1993, while the EBI and PIR both developed their own sites for UniProt, with a similar appearance, but different code and functionality. Though the redirection was done according to the geographic location of the client, it happened occasionally that users were confronted with a site that looked and worked differently from the one they were used to. To provide users with a consistent view and to cut the cost of maintaining three separate sites, the consortium decided to develop a common website for UniProt. Following several years of intense development and a year of public beta testing, the  domain was switched to the newly developed site described in this paper in July 2008.

### Requirements

The essential functionality that the website (like its predecessors) had to provide was:

• Retrieval of individual database entries by identifier.

• Retrieval of sets of entries based on simple search criteria such as organism, keyword or free text matches.

• Display of data in a human readable manner.

• Download of data in all official formats.

• Basic tools for identifier mapping, sequence alignments and similarity searches.

• Access to documentation and controlled vocabularies.

An additional wish was that each consortium member should be able to host a mirror of the website without too much effort, and that the technology on which the website was to be built should be familiar enough to allow all consortium members to contribute to the development. Beyond that there was no shortage of ideas for bells and whistles, such as data mining and visualization tools. However, a careful review of the archived help desk questions and web server request logs, collected over several years from the existing sites, revealed the following:

• The majority of the queries consisted of nothing more than a protein or gene name, sometimes combined with an organism name. Some of these queries did not yield useful results, because of the lack of a good scoring algorithm (e.g. searching for "human insulin" could require scrolling through hundreds of results before finding the most relevant entries, such as INS_HUMAN).

• Some queries yielded no results because people misspelled terms or did not use the same conventions as UniProt (e.g. American vs English spelling, Roman vs Arabic numbers in protein names, dashes vs separated words) or chose the wrong field in an "advanced" search form, etc. Some of this was documented, but the documentation was not accessed much.

• The majority of requests came from web crawlers and other automated applications (many of which made valid use of our data). Referrals from search engines made up a substantial part of the visits, therefore we did not want to block web crawlers either, yet this was putting quite a bit of a load on our servers.

Ensuring that these issues would be resolved by the new site, along with all the basic requirements, was therefore made a priority [2].

## Construction, content and utility

### What data is available on the site?

The UniProt web site provides access to the data sets presented in Table 1.

**Table 1:** Overview of the UniProt data sets

**Data set**	**Description**	**References**	**Entries**	**Path**	**Formats**
UniProtKB	Protein sequence and annotation data	UniRef, UniParc, Literature citations, Taxonomy, Keywords	6.4 M	/uniprot/	Plain text, FASTA, (GFF), XML, RDF
UniRef	Clusters of proteins with similar sequences	UniProtKB, UniParc, Taxonomy	12.3 M	/uniref/	FASTA, XML, RDF
UniParc	Protein sequence archive	UniProtKB, Taxonomy	17.0 M	/uniparc/	FASTA, XML, RDF
Literature citations	Literature cited in UniProtKB (based on PubMed)		0.4 M	/citations/	RDF
Taxonomy	Taxonomy data (based on NCBI taxonomy)		0.5 M	/taxonomy/	RDF, (Tab-delimited)
Keywords	Keywords used in UniProtKB		1K	/keywords/	RDF, (OBO)
Subcellular locations	Subcellular location terms used in UniProtKB		375	/locations/	RDF, (OBO)

### How is the site structured?

The pattern for URL templates shown in Table 2 is used not only for the main data sets, but also for the various "ontologies", for documentation and even running or completed jobs.

**Table 2:** URL templates

**Template**	**Description**	**Example**
http://www.uniprot.org/{dataset}/	Overview page for a data set, may contain a description of the data set along with various entry points, or just list all database items (equivalent to searching for *).	http://www.uniprot.org/uniprot/
http://www.uniprot.org/{dataset}/?query={query}	Filters the data set with the specific query. Other parameters are "offset" (index of first result), "limit" (number of results to return), "format" (e.g. "tab" for tab-delimited or "rdf") and "compress" ("yes" to gzip results when downloading).	http://www.uniprot.org/uniprot/?query=green
http://www.uniprot.org/{dataset}/{id}	Displays a specific database entry.	http://www.uniprot.org/uniprot/P00750
http://www.uniprot.org/{dataset}/{id}.{format}	Returns a database entry in the specified format.	http://www.uniprot.org/uniprot/P00750.rdf

There are no special search pages. The search function and other tools can be accessed directly through a tool bar that appears at the top of every page. Depending on the current context, some of the tool forms are pre-filled. For example, when viewing a UniProtKB entry, the sequence search form is pre-filled with the sequence of the entry, and the alignment form is pre-filled with all alternative products of the entry, if any.

### How to get people started?

**Figure 1:**
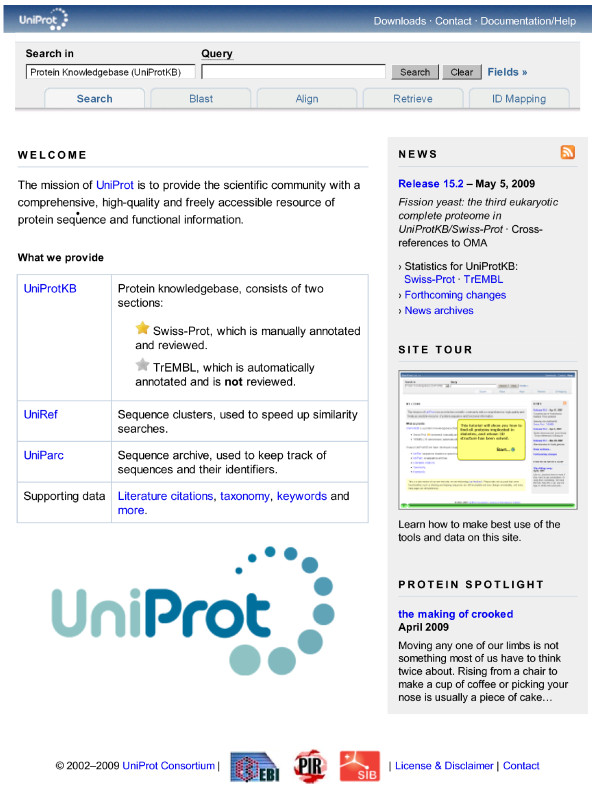
**Home page at **.

Important information is often overlooked on home pages with a lot of content. The new UniProt home page (see Figure 1) features a prominent tools bar, that is present on every page and serves as a basic site map with links to common entry points. The site contains a lot of small, useful features that are documented in the on-line help; however, people in general appear to be reluctant to invest a lot of time into reading documentation. To address this issue, we recorded a "site tour" [3] that is accessible from the home page.

### How to get the text search function right?

The text search function is the most used feature on the website. Considerable effort was therefore invested into making all common and less common searches not only possible, but also simple and convenient to use for people without a detailed understanding of UniProt data. One of the most obvious problems with the old sites had been the lack of good relevance scoring of search results. Scoring is essential for queries that are meant to locate specific entries, but that contain terms that appear in a large number of entries (e.g. the "human insulin" example quoted above). The main factors that influence the score of an entry for a given query on the new website are:

• How often a search term occurs in an entry (without normalizing by document size, as this would benefit poorly annotated documents).

• What fields in an entry a term occurs in (e.g. matches in a protein name are more relevant than in the title of a referenced publication).

• Whether an entry has been reviewed (reviewed entries are more likely to contain correct and relevant information).

• How comprehensively annotated an entry is (all else being equal, we want to have a bias towards well-annotated entries).

The exact scoring scheme differs for each data set and requires ongoing fine-tuning.

**Figure 2:**
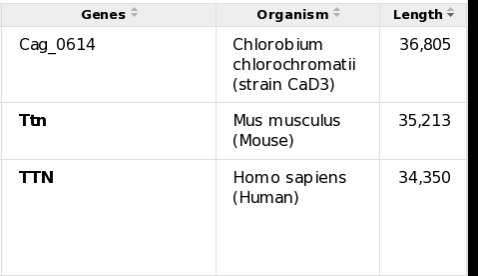
**UniProtKB search results, sorted by length descending**.

In order to allow people to see quickly the entries with e.g. the longest or shortest sequences, or to page through the results one organism at a time, certain fields were made sortable. This turned out to be not trivial to implement as the underlying search engine library had no support for sorting results efficiently on anything but their score. Therefore, special sort indexes are now built when the data is loaded, at the cost of slowing down incremental updates. Figure 2 shows the result of a query in UniProtKB, sorted by length descending.

The traditional approach of having two separate forms for "basic" and "advanced" queries has several issues: Based on our observations, few people start out with the intention of using an "advanced" form. Even if they have a good understanding of the data and search function, they often first try to obtain the results through a simple search form, as these are quicker to fill in. If the basic search does not yield the expected result, or too many results, the query has to be redone in an advanced search form where further constraints can be applied.

Another problem with "advanced" search forms is that they often do not take into account that most nontrivial queries appear to be built iteratively: People start out with one or two terms, and then add or modify (e.g. constrain) terms until they see the desired results or give up. If multiple complex constraints are specified at once and the query produces no results, it can be time-consuming to figure out which (if any) of the constraints was used incorrectly. We therefore opted for a "fail early" approach: A simple full text search is the fastest and most effective way to determine whether or not a term even appears in a database, as you can skip the step of scrolling through and selecting a field, and then having to wonder if the term might have appeared in another field.

**Figure 3:**
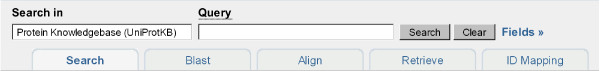
**Basic search form**.

For these reasons, we opted for a single search form(see Figure 3). People start by searching for one or two terms. The results page shows the matches for these terms and, for people who are not familiar with our search fields, clickable suggestions such as:

• "Did you mean" spelling suggestions (if there are no or few results and the index contains a similar word).

• Restrict a term to field (listing only fields in which a term occurs).

• Quote terms (if they frequently appear together in the index).

• Filter out unreviewed or obsolete entries (if the results contain such entries).

• Replace a field with a more stringent field (if this helps reduce the number of results).

• Restrict the range of values in a field (if results are being sorted on this field).

• ...and others, depending on the context.

This approach allows people to move seamlessly from a basic to an advanced query without prior knowledge of the fields used to store data in UniProt. Clicking on a suggestion requires less mouse clicks than selecting a field in an "advanced" search form. It is also more effective, because only the fields in which a term occurs are listed – such a filtering is difficult to accomplish with traditional "advanced" search forms.

**Figure 4:**
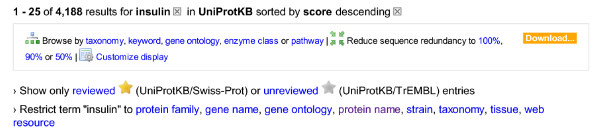
**Suggestions for a simple query, **.

Figure 4 shows suggestions for a simple query, .

Each step in the query building process updates the query string and is reflected in the URL, so it can be bookmarked, or undone by hitting the back button. The step-by-step process does not preclude expert users entering complex queries directly, which can be faster and more powerful (e.g. Boolean operators) than using an "advanced" query form. Table 3 provides an overview of the query syntax.

**Table 3:** Query syntax overview

**Query**	**Returns**
human antigen	All entries containing both terms
human AND antigen	

"human antigen"	All entries containing both terms in the same order

anti*	All entries containing terms starting with anti. To search for a term that contains an actual asterisk, escape the asterisk with a backslash (anti\*). Asterisks can be used within and at the end of terms.

human-antigen	All entries containing the term human but not antigen
human NOT antigen	

human OR antigen	All entries containing either term

antigen (human OR pig)	Using brackets to override Boolean precedence rules

author:Tiger*	All entries with a citation that has an author whose name starts with Tiger. Note the field prefix author; had we left it out, there would have been a large amount of unwanted results.

gene:L\(1\)2CB	All entries with the specified gene name. Note how the backslash is used to escape the brackets, which would otherwise be interpreted as part of a Boolean query. Other characters that must be escaped are: []{}?:~*

gene:*	All entries that have a gene name.

A frequent cause of failed queries in past implementations were trivial differences such as the use of dashes (e.g. "CapZ-Beta" vs "CapZ Beta") or Roman vs Arabic numbers in names (e.g. "protein IV" vs "protein 4"). Such cases are now treated as equivalent. Many search engines stem words, for example they would treat the search terms: "inhibit", "inhibits" and "inhibiting" as equivalent. However, given that most of the queries consisted of names, such as protein, gene or organism names, where such stemming is dangerous, and that there is no way to know whether or not an entered term should be stemmed, this was left out.

**Figure 5:**
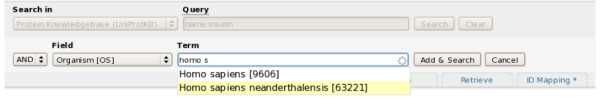
**Using the query builder to add a constraint**.

One advantage of "advanced" search forms is that they allow to present fields that have a limited number of possible values as drop-down lists or offer auto-completion, if the field contains values from a medium- to large-sized ontology. This functionality can, however, also be integrated into a "simple" search form: We chose to provide the possibility to search in specific fields of a data set by adding one field search constraint at a time. The user clicks on "Fields >>", selects the desired field and enters a value and then clicks "Add & Search" to execute the query. Further search constraints can be added to refine the query iteratively until the desired results are obtained (see Figure 5).

Certain data sets reference each other: This can be used to do subqueries, e.g. while searching UniRef you can add a constraint "uniprot:(keyword:antigen organism:9606)" to show only UniRef entries that reference a UniProt entry with the specified keyword and organism. This functionality can sometimes also be accessed from search results, e.g. while searching UniProtKB there may be a "Reduce sequence redundancy" link that converts the current query into a subquery in UniRef.

The search result table of most data sets can be customized in two ways: The number of rows shown per page can be changed, and different columns can be selected.

Note that the choice of columns is preserved when downloading the results in tab-delimited format.

**Figure 6:**
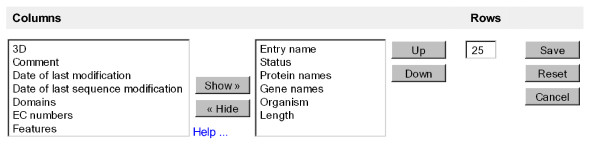
**"Customize display" option for UniProtKB search results**.

Figure 6 shows a screenshot of the "Customize display" option for UniProtKB search results.

### How to support download of custom data sets?

We receive frequent demands to provide various downloadable entry sets, such as all reviewed human entries in FASTA format. While some of the most frequently requested files can be distributed through our FTP server, doing so is obviously not feasible for many requests (especially for incremental updates such as all reviewed, human entries in FASTA format added or updated since the beginning of this year). Such sets can now be obtained from the website, which no longer imposes any download limits. However, large downloads are given low priority in order to ensure that they do not interfere with interactive queries, and they can therefore be slow compared to downloads from the UniProt FTP server.

### How to support browsing?

**Figure 7:**
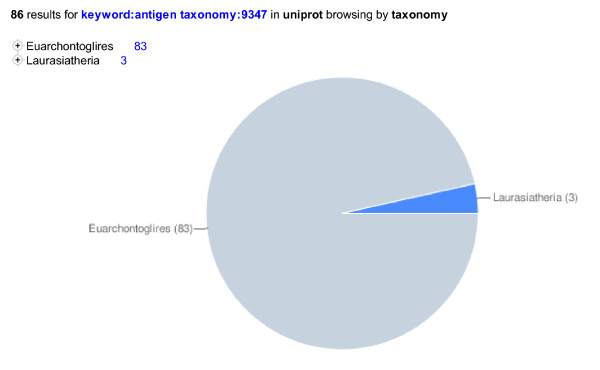
**Using hierarchical collections to browse search results**.

The two main modes of looking for data are 1. with direct searches and 2. by browsing, i.e. following links through a hierarchical organization. The new website makes use of various ontologies (Taxonomy, Keywords, Subcellular locations, Enzyme, Gene Ontology, UniPathway) to allow users to browse the data or combine searching with browsing (e.g. search for keyword:Antigen and then browse by taxonomy, see Figure 7).

### How to allow selection of multiple items?

**Figure 8:**
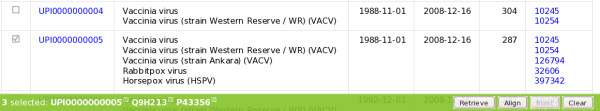
**Using the cart to select items across multiple data sets**.

Using a list of results will often imply performing further action, such as downloading all or selected items, or aligning corresponding sequences. The simplest solution would be to add check boxes next to the items and enclose them in a form that also contains a list of tools to which the items can be submitted. The problem with this approach is that it can result in some redundancy in the user interface: when adding a tool, it is necessary to add it everywhere where items can be selected. Moreover, this approach does not allow selection of items across multiple pages (e.g. when paging through search results) or across different queries or data sets. The solution that was implemented was to provide a general selection mechanism that stores items in a "cart". The contents of the cart are stored as a cookie in the web browser (so it does not require any state to be stored on the server side). The cart itself has certain actions attached to it such as "Retrieve" or "Align", and can be cleared with a single click. As shown in Figure 8, the cart also allows to select items across multiple data sets.

### How to show complex entries?

The most important data on this site can be found in UniProtKB, in particular in the reviewed UniProtKB/Swiss-Prot entries. These entries often contain a large amount of information that needs to be shown in a way that allows easy scanning and reading:

• Names and origin

• Protein attributes

• General annotation (Comments)

• Ontologies (Keywords and Gene Ontology)

• Binary interactions

• Alternative products

• Sequence annotation (Features)

• Sequences

• References

• Web resources (Links to Wikipedia and other online resources)

• Cross-references

• Entry information (Meta data including release dates and version numbers)

• Relevant documents (List of documents that reference an entry)

Describing the information found in UniProtKB/Swiss-Prot [4] is outside the scope of this paper. Here are some improvements that were made over previous attempts to show this data:

• Features and cross-references are categorized.

• Features have a simple graphical representation to facilitate a comparison of their locations and extents.

• Secondary structure features are collapsed into a single graphic.

• Alternative products are listed explicitly in the "sequences" section.

• Sections can be reordered or hidden (and these changes are remembered).

**Figure 9:**
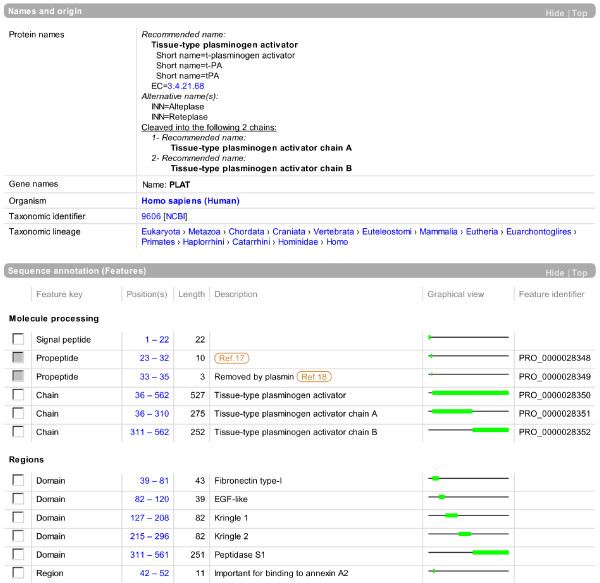
**Parts of two sections from the UniProtKB entry view shown at **.

Parts of two sections from the UniProtKB entry view of human tissue-type plasminogen activator (P00750) are shown in Figure 9.

### How to integrate sequence similarity searches?

**Figure 10:**
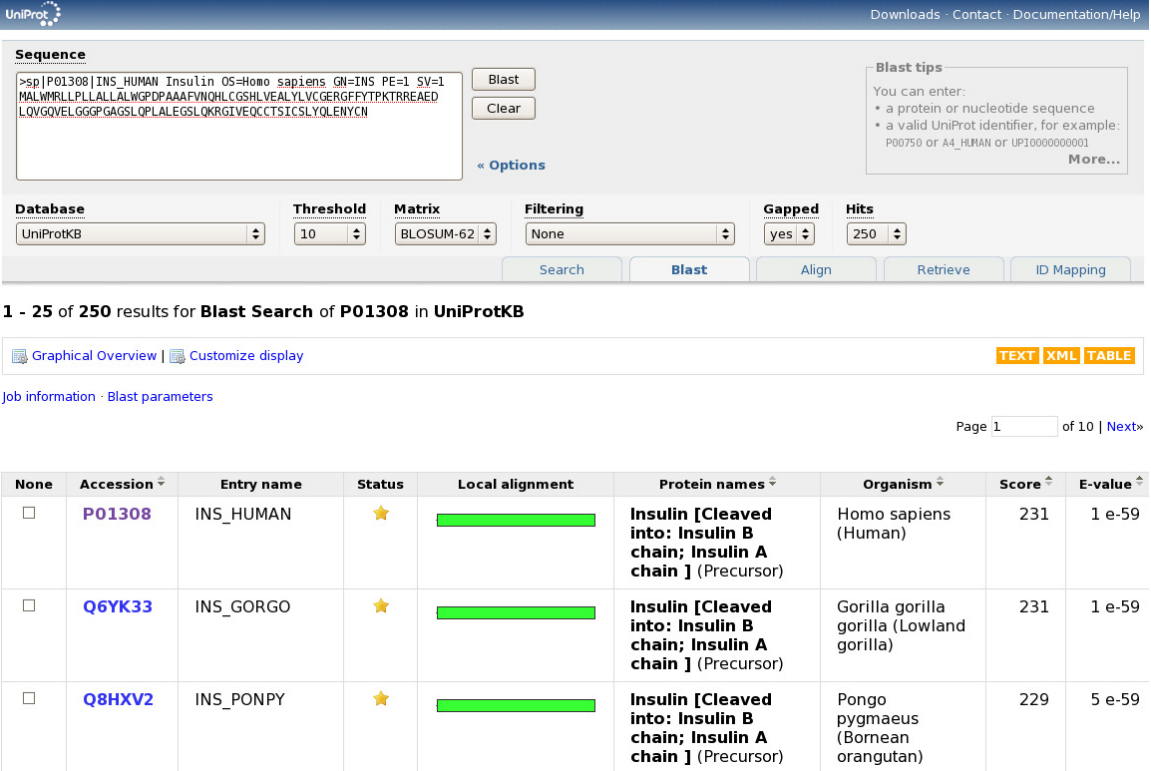
**Sequence similarity search form and results**.

In addition to text searches, sequence similarity searches are a commonly used way to search in UniProt. They can be launched by submitting a sequence in FASTA format, or a UniProt identifier, in the "Blast" form of the tools bar. Note that this form is pre-filled with the current sequence when viewing a UniProtKB, UniRef or UniParc entry. Figure 10 shows the sequence similarity search form and results.

### How to integrate multiple sequence alignments?

**Figure 11:**
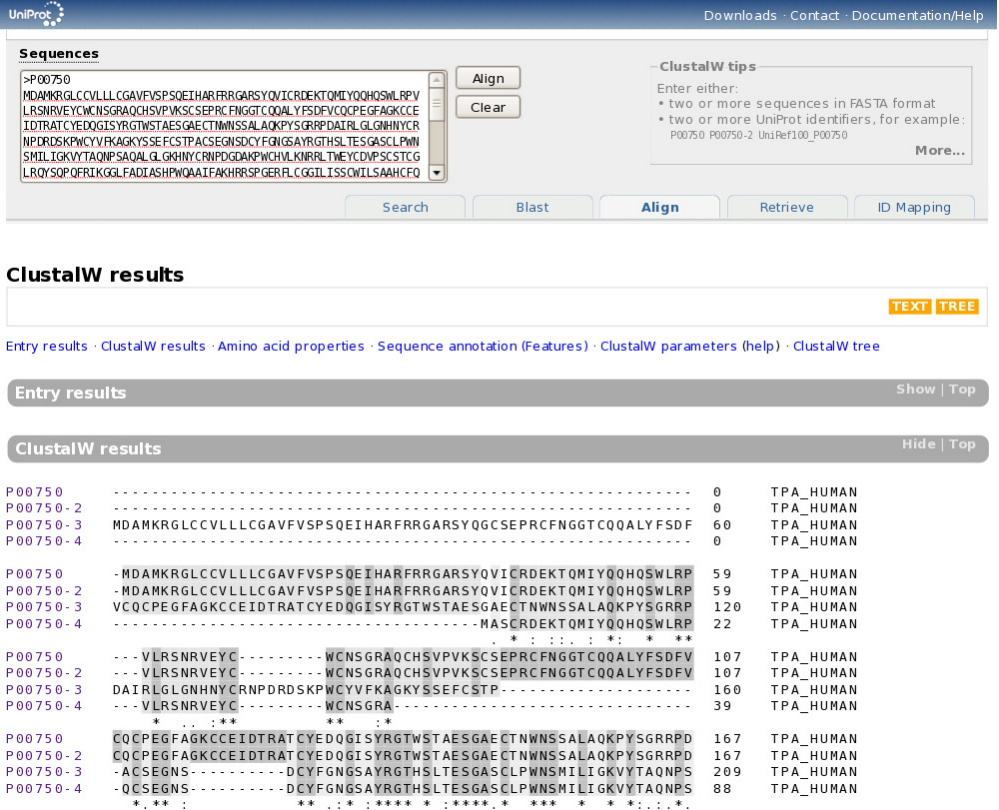
**Multiple sequence alignment form and results**.

The purpose of the integrated "Align" tool is to allow simple and convenient sequence alignments. ClustalW is used because it is still the most widely used tool, though it may no longer be the best-performing tool in all cases. The form can be submitted with a set of sequences in FASTA format or a list of UniProt identifiers, or more likely through the built-in "cart". The form is pre-filled with a list of sequences when viewing a UniProtKB entry with alternative products or a UniRef cluster. For complex alignments that require specific options or a specific tool, the sequences can easily be exported into FASTA format for use with an external alignment tool. Figure 11 shows the multiple sequence alignment form and results.

### How to integrate identifier mapping functionality?

**Figure 12:**
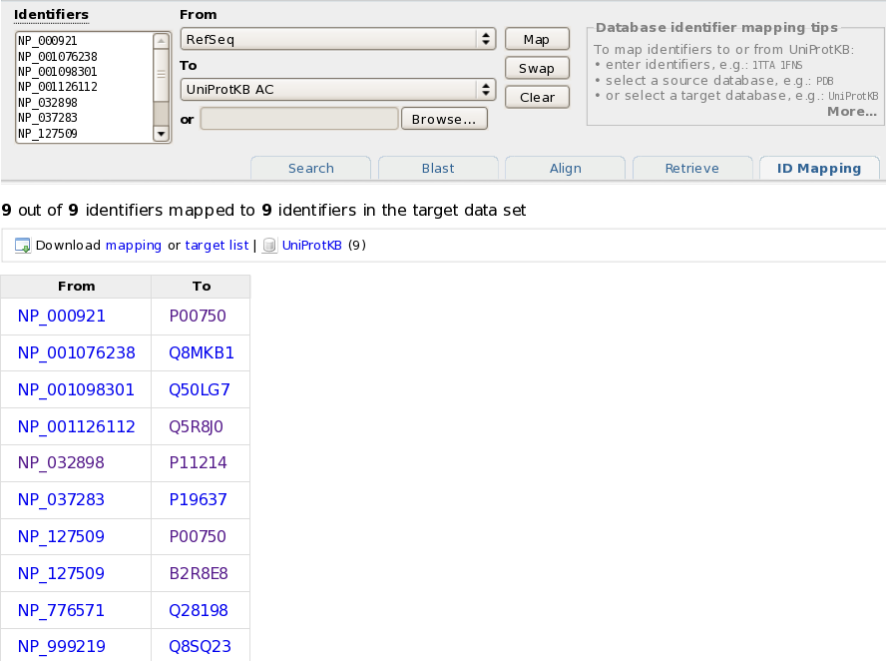
**Mapping RefSeq identifiers to UniProtKB**.

There is an identifier mapping tool that takes a list of UniProt identifiers as input and maps them to identifiers in a database referenced from UniProt, or vice versa. An additional supported data set that can be mapped is NCBI GI numbers. Figure 12 shows how RefSeq identifiers can be mapped to UniProtKB.

### How to retrieve UniProt entries in batch?

**Figure 13:**
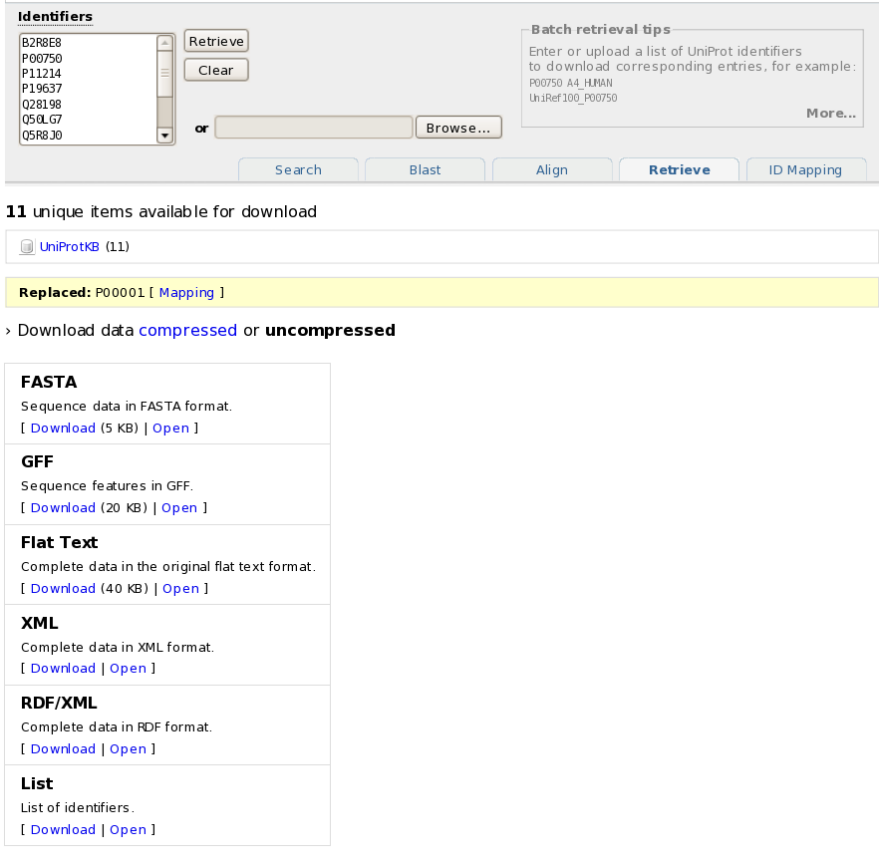
**Batch retrieval of a set of UniProtKB entries**.

The batch retrieval tool allows to specify or upload a list of UniProt identifiers to retrieve the corresponding entries (see Figure 13). The available download formats are the greatest common denominator: For example, if the batch retrieval request contains both UniProtKB and UniParc identifiers, neither plain text (only available for UniProtKB) nor XML (available for both, but with different schemas) will be available, only FASTA and RDF. The set of entries retrieved by their identifiers can then optionally be queried further, using the search engine described previously.

### How to handle job submissions?

**Figure 14:**
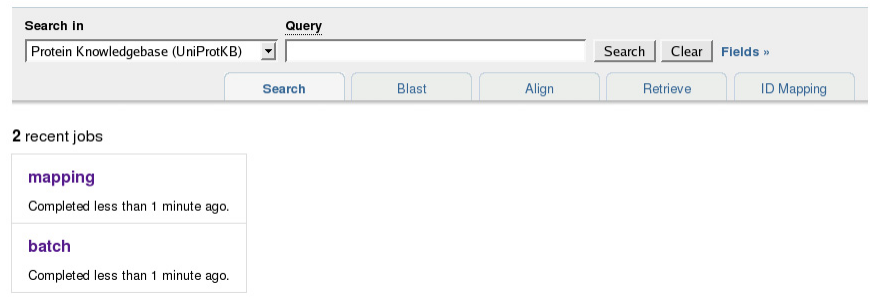
**Recent jobs run by the current user, shown at **.

The website is a read-only, stateless application, with the exception of the job handling system. When e.g. a database mapping job is submitted, a new "job" resource is created, and the user is redirected to this job page (which will initially just show the job status, later the results). A consequence of this is that if a web server receives a request for a job that it does not have, it needs to ask all other mirrors if they have this job (and if yes, transfer it). Jobs have unique identifiers, which (depending on the job type) can be used in queries (e.g. to get the intersection of two sequence similarity searches). Recent jobs run by the current user can be listed using the URL  (see Figure 14).

### How to deal with web crawlers?

Search engines are an important source of traffic, perhaps because people now tend to "google" search terms before trying more specialized sites. Therefore it is important to ensure that search engines are able to index (and keep up to date) as much content of the site as possible. However, web crawlers have trouble finding pages, such as database entries, that are part of large collections and are not linked from main navigation pages, and when they do, this can put a significant load on the site. To ensure that web crawlers find all content that was meant to be indexed, the content was linked from multiple sources, including overview documents and machine-readable site maps [5]. To keep web crawlers away from content that is either not worth indexing or too expensive to retrieve on a large scale, a robots.txt file [6] is used, and links that should not be followed were marked with a "nofollow" "rel" attribute [7]. The retrieval performance of documents in large collections was optimized until we felt confident that even rapid crawling would not impact the overall responsiveness of the site too much. Such documents also return a "Last-modified" date header when requested. Certain web crawlers (e.g. Googlebot) will then on their next visit issue a conditional "If-Modified-Since" request, so there is no need to resend unchanged documents. Since each resource can now be accessed through one URL only, there is no more redundant crawling of resources (as used to be the case with multiple mirrors with different addresses). The request logs and "Google Webmaster Tools" site [8] are used to monitor the behavior of web crawlers on this site. As of July 2008, over 4 M pages from the new site are indexed in Google.

### How to avoid breaking links?

This site publishes a large number of resources (several million) on the Web. These resources are linked from a lot of other life sciences databases as well as scientific papers. Since tracking down and getting all such links updated is not practical, and keeping legacy URL redirection schemes in place for a long time can be tedious, it is worth investing some effort into reducing the likelihood that large sets of URLs will have to be changed in future [9]. Technology artifacts, such as "/cgi-bin/" or ".do", are avoided in URLs [10]. Official and stable URLs are no good if they are not used. A lesson learned from the previous sites was that the URLs that end up being used are those that are shown in the browser (i.e. mirror-site specific URLs). The new site avoids this problem by having exactly one URL for each resource. Another issue is how to deal with individual resources that are removed. Obsolete entries, or entries that were merged with other entries, in the main data set (UniProtKB) no longer disappear from the web interface, but keep their own web page (e.g. ), with a link to a list of (retrievable) previous versions (e.g. ). Specific versions can also be referenced directly (e.g. ), and used in the tool forms (e.g. P00001.48).

### How to support user-defined customizations?

Some simple customizations, such as being able to choose the columns shown in search results, make the site a lot more convenient to use. However, we did not want to compromise the statelessness of the application (which is important for keeping the application distributable and scalable) by having each request depend on centralized user profile data. This was possible by storing basic settings in client-side cookies. The drawback of this solution is that such settings are lost when cookies are cleared in the browser or when the user switches to another machine. The amount of data that can be stored this way is also limited. On the other hand, the customizations are simple and easy to redo. This solution also does not require people to sign up, which some may be reluctant to do as this introduces some privacy issues.

### How to let people tailor the web site to their needs?

The ideal site would be a "one stop shop" that solves all our users' needs. However, the life sciences community has very diverse needs, and our data is often just one small part of these needs. The best we can do is make it as easy as possible to retrieve data from this site programmatically, in order to facilitate the development of applications on top of our data.

### How to enable programmatic access to the site?

People need to be able to retrieve individual entries or sets of entries in various formats and use our tools simply and efficiently from within basic scripts or complex applications. We want to encourage people to build applications that are tailored towards certain user communities on top of our data – customization options on our site can go only so far, and anything we build will always be focused on our data.

Early versions of the new site had a complete SOAP [11] interface built with Apache Axis [12]. Unfortunately this interface had poor performance (which necessitated introducing limitations such as the maximum number of entries that could be retrieved in one go), and simple operations such as retrieving an entry in FASTA format ended up being more complex than could be justified. For example, in order to retrieve the data from a Perl script, a special module (SOAP::Lite) had to be installed and patched, due to quirks with the support for SOAP attachments. Meanwhile, there are better SOAP libraries, but they are still more complicated and less efficient to use than doing direct HTTP requests.

To ensure that such "RESTful" [13] access is as simple and robust as possible, the site has a simple and consistent URL schema (explained in a previous section) and returns appropriate content type headers (e.g. application/xml for XML resources) and response codes. Returning appropriate HTTP status codes instead of returning 200 OK for all requests, even if they fail, has several benefits:

• Ensures that invalid pages stay out of search engine indexes

• Simplifies error handling for people doing programmatic access (no need to have fragile checks for error message strings)

• Helps detect common problems when analyzing request logs.

Table 4 lists all response codes that the site might return. The most important distinction are the 4xx response codes, which indicate that there is some problem with the request itself and sending the same request again will likely fail again, and the 5xx response codes, which indicate a problem with the server.

**Table 4:** Listing of response codes that the site might return

**Code**	**Description**
200	The request was processed successfully.
301	Moved (permanently). Use the new address for future requests
302	Moved (temporarily)
400	Bad request. There is a problem with your input.
404	Not found. The resource you requested doesn't exist.
410	Gone. The resource you requested was removed.
500	Internal server error. Most likely a temporary problem, but if the problem persists please contact the site operators.
503	Service not available. The server is being updated, try again later.

In addition to the data set-dependent formats, all search results can be retrieved as OpenSearch [14] RSS feeds for integration with external tools such as news feed readers or Yahoo Pipes [15].

### How to handle complex data?

While the needs of most people are met by being able to obtain data in FASTA, tab-delimited or even plain text format, some people need to obtain and work with the complete structure of the data. This is complicated by the fact that the data model is complex and changes a lot. RDF (part of the W3C's Semantic Web initiative [16]) provides a generic graph-like data model that can help address these issues [17]. All UniProt data is available in RDF as well as XML formats both on our FTP servers (for bulk downloads) and on the site.

Resources in RDF are identified with URIs. UniProt uses PURLs [18]. For example,  is used to reference and identify the concept of the human taxon. This URL can be resolved to either  (human readable representation; returned e.g. when entered in a browser) or  (machine-readable representation; returned if the request contains an "Accept: application/rdf+xml" header).

### What is the architecture of the site?

The website was implemented as a pure Java web application that can either be run standalone using an embedded web server, Jetty [19], or deployed on any Servlet 2.4 compliant [20] web application server. The components in the application are configured and connected together using the Spring Application Framework [21]. Struts [22] coordinates the request handling, and pages are rendered as XHTML using JSP (2.0) templates. Database entries are stored in Berkeley DB JE [23] for fast retrieval. Searching was implemented with help of the Lucene text search library [24].

Spring was introduced to remove hard-coded dependencies (or hard-coded service lookups) from the code, as this was hampering our ability to unit-test the code. Struts was chosen among the plethora of available web application frameworks because it provided some conveniences, such as the automatic population of objects from request parameters, but did not attempt to abstract too much (e.g. we needed access to the HTTP request and response objects in order to read and set certain HTTP headers). Struts is (or was) also a de facto standard and simple to learn. Using Berkeley DB JE to store serialized Java objects using custom serialization code was by far the most efficient solution for retrieving data that we tested. Extracting data from uncompressed text files using stored offsets might be faster for returning data in specific formats. However, the size of the uncompressed files and number of different databases and formats make this approach less practical than generating the various representations on the fly. Minimizing the amount of data that is stored on disk also ensures that the application can benefit more from increasing disk cache sizes.

The website application is self-contained and can be run out of the box with zero configuration for development and test purposes. Non-Java and compute-intensive tools such as sequence similarity searches (BLAST), multiple sequence alignments (ClustalW) and database identifier mappings are run on external servers. To minimize the footprint of the application further, historical data such as entry versions from UniSave [25] is also retrieved remotely on demand. Even so, the data including indexes occupies almost 140 GB (as of release 14.5). For development and testing, a smaller, internally consistent, data set (~2 GB) is generated by issuing queries to a site that has the complete data loaded.

### How to deploy the site on distributed mirrors?

With the previous setup, each "mirror" site had its own public address. Requests to  were redirected to  (EBI),  (PIR) or  (SIB), respectively. The redirection was done with client-side HTTP redirects and was based on the top level domain (TLD) revealed by a reverse DNS lookup of the IP address from which a request originated. One major problem with this setup was that people and web crawlers bookmarked and linked to the mirror they had been redirected to rather than the main site. Tracking down such links and getting them corrected turned out to be a Sisyphean task. One consequence was that people more often than not ended up neither on the nearest mirror, nor did they get the benefits of failover. The new setup makes use of the fact that you can attach multiple IP addresses (A records) to a domain name. Clients will connect more or less randomly to one address that is reachable. We also tested whether it was possible to achieve geographic affinity by having different name servers return different IP addresses depending on which mirror was nearer. This can work because the "caching" name servers that clients must use to resolve addresses often keep track of what resolving name server responds faster (and therefore is most likely the nearest) for a given domain. The tests showed that this worked to some degree, at least for the most frequent users. The drawback of this solution is that with only two mirrors, no failover is possible when one of the sites happens to become unreachable. Given that network delays between the U.S. and Europe are not too bad, reliability was seen as more important. This may change if one or more additional mirrors are set up in more remote places.

The current mirror sites deploy the web application on Tomcat [26] and use Apache [27] as a reverse proxy [28], as well as for request logging, caching and compressing responses (the latter can have a huge impact on page load times for clients on slow connections). If the application is not available at one site (e.g. while it is being updated), Apache automatically sends requests to another available mirror. The web application has a special health-check page that is monitored from local scripts (which notify the local site administrators if there is a problem), as well as from a commercial monitoring service. This service can also detect network problems and keeps statistics on the overall reliability and responsiveness of each mirror. Application-level warnings and errors are handled by Log4j [29]. Errors trigger notification messages that go directly to an e-mail account that is monitored by the developers (unless an error was triggered by a serious operational issue, it usually indicates a bug in the code). Finally, there is a JMX interface that supplements JVM-level information, such as memory use, and Tomcat-supplied information, such as the number of open HTTP connections, with application information, such as the hit ratios of specific object caches.

### How to manage data and application updates?

Data can be loaded into the web application simply by dropping a data set, or partial data set for incremental updates, in RDF format into a special directory and waiting for the application to pick up and load the data. However, to save resources, we load all the data on a single staging server and then distribute the zipped data to the mirror sites, usually along with the latest version of the web application. Updates occur every three weeks, in sync with the UniProt releases.

### How to reduce the risk that bugs are introduced into the code?

All code changes risk introducing bugs, which can be time-consuming to fix, especially when not detected right away. To minimize this risk, automated tests need to be set up. The lowest-level testing is done in the form of "unit" tests. The goal of unit tests is to cover each execution path in the code using different input. Single classes are tested in isolation. Such tests were written using the JUnit testing framework [30]. The initial test coverage was quite low, as there was no simple way to untangle classes for isolated tests. This was improved by introducing a "dependency injection" framework [21] to remove hard-coded dependencies. Unit tests are complemented with "functional" tests. We created a set of test scripts with the open source tool Selenium [31]. These tests can be played back in any recent browser and simulate typical user interactions with the site. Reducing the playback speed allows semi-automatic testing, where a person watches the test execution to catch layout glitches that would be difficult to catch with fully automated tests. In addition to testing the site prior to releases, these tests can be used for browser compatibility testing. We attempted to ensure that the site works well with the most popular browser and operating system combinations (e.g. Internet Explorer 6 and 7 on Windows, Firefox 2 on Linux) and acceptably with other, recent browsers (e.g. Safari on Mac OS X). Another major "functional" test is loading all data into the site. Given that this is a long procedure, a smaller test data set is often used instead to verify that the import procedure is working. Other one-off tests included setting up and using a tool to compare search results returned by the new and the old sites.

### How to ensure that the site will have adequate performance?

Basic performance goals were established based on numbers obtained from the request logs of the previous sites. Even though the application is stateless (i.e. no state is stored on the server) and can therefore be scaled out horizontally (i.e. by buying more machines), the stated goal was to be able to support the full load on a single, powerful, but "off-the shelf" machine. Following are some load tests that were performed to identify potential issues and help build confidence in the application:

• Retrieve random database entries

• Execute random queries

• Download large result sets

• Simulate initial requests to the home page including all required static resources

The tests were performed with a combination of shell scripts and the httperf tool [32]. Performance numbers, such as response times, were analyzed with R [33]. Once a performance issue was found, problematic code was tracked down with the help of a commercial profiling tool [34]. Performance issues on the production servers are caught 1. by an external monitoring tool that records response times for requests to a general health-check page, and 2. by analyzing the request logs (which include the duration of each request) at the end of each month. While the former allows immediate action to be taken, the latter can help to detect more subtle performance issues.

### How to ensure that people will know how use the site?

For all but the most trivial functions it is difficult to predict whether people will be able to figure out how to use them. The most effective way to answer such questions is to do "usability" tests [35]. We managed to recruit a dozen or so volunteers who let us watch them use the site to accomplish certain tasks. Most of them had some kind of life sciences background, however, not all of them had been working with our data on a regular basis. The tests took on the following form: Two people, one to ask questions, the other to take notes, went to the volunteer's workplace (if possible; this ensured that people had the setup they were used to and felt comfortable). Some brief background questions were asked to establish how familiar the person already was with our data, what services and tools they had used in the past, etc. Based on this information they would then be asked to accomplish certain tasks on the site. We tried as best as possible to avoid putting the user under pressure. The other difficulty was avoiding phrasing tasks in terms of the concepts we were using. For example, when asked to fill out the "contact form", people would immediately find the "contact" link. But if we phrased the question in different terms, such as "send feedback", success was less guaranteed. Testing sessions were between half an hour and one hour and helped settle and open up quite a few "design" discussions.

### How to keep track of what is being used?

In order to know how the site is being used, and which parts of the site are working well and which are not, it is essential to collect data on the site's usage. Usability testing is invaluable and can help pinpoint certain issues, but it is time-consuming and therefore unsuitable to collect large sample sizes. Fortunately, some basic information on user interactions with the site can be recorded through the web server log files. In addition to the standard information logged for each request (such as the exact time and path of the request, the response code, the IP address, referrer and user agent), the web server was configured to also record the duration of the request, the number of search results returned (if the request was a query) and the content-type of the response (this could often be inferred from the extension of the requested resource, but not always). The request logs are collected from all mirror sites once a month, cleaned up a bit and loaded into a simple star schema in a relational database. This allows us not only to get general usage statistics (e.g. total number of requests for different resources, showing the percentage of automated requests), but also to look for problems (e.g. queries that do not produce any results or fail) and even help to set annotation priorities (e.g. by looking at what are the most frequently requested entries that have not been reviewed yet, or not updated in a long time).

Another complementary tool that is used to gather statistics is Google Analytics [36], which records data through JavaScript embedded in the web pages. The advantage and drawback of this approach is that it does not record automated requests, such as those issued by web crawlers, or requests to non-HTML resources. While Google Analytics was left enabled during all of the beta phase, it is now only enabled from time to time. We use it to check the (less accurate) number of non-robot requests reported via the request log analysis procedure, which relies on user agent strings matches and the patterns need to be updated from time to time. It also helps us to get an idea of the browsers and screen resolutions people are using, information which is less accurate or impossible to get via the web server request logs. Google Analytics can provide fast and convenient feedback on the impact of certain changes, as data is updated at least once a day, but it can also slow down the perceived page loading time and aggravate certain privacy concerns.

### How to "go live" with minimal casualties?

Despite all the load and usability testing, there was no guarantee that switching over all the old sites to the new site in one go would not swamp us with more technical issues and irate users than we could possibly handle at once. Having a prolonged "beta" period allowed us to get feedback from many users and to ramp up the number of people (and web crawlers, etc) using the new site gradually by taking these steps (moving to the next step whenever we felt comfortable enough to do so):

1. Sending out invitations to certain people and groups to use the new site.

2. Getting the site indexed in Google.

3. Linking the old sites to the new site.

4. Switching over the old sites one by one.

## Discussion

Collecting data and optimizing the use and performance of the website is an ongoing process.

One of the biggest challenges UniProt is facing is getting more community involvement to help cope with the increasing amount and complexity of data. Simply having more people give feedback when they see incorrect or missing data in UniProt would already be a huge improvement. One possible approach under investigation is to make such feedback more rewarding by replacing or supplementing the conventional feedback forms with a commenting system. Other more complex approaches, such as using wiki software, are under investigation as well [37].

Looking beyond UniProt: Much of the development effort was spent on issues that are not UniProt-specific, ranging from handling identifiers in a stable way to most of the code for the search engine. These issues are likely to be relevant for other life sciences databases as well. Had there been some kind of framework that provided these features, the development time could have been reduced significantly. This seems especially important for smaller databases that may not have resources to reinvent the wheel. It may therefore be worth incorporating some of the solutions we have come up with here into a framework (or add them to existing frameworks).

## Conclusion

The new UniProt website makes accessing and understanding UniProt easier than ever. The two main lessons learned are that 1. getting the basics right for such a data provider website (and likely others as well) has huge benefits, but is not trivial and easy to underestimate, and that 2. there is no substitute for using empirical data throughout the development process to decide on what is and what is not working for your users. We hope to encourage more people in the life sciences community to resist the temptation to spend time adding bells and whistles to an application before getting the basics right, and to put in place rigorous procedures for assessing whether or not the site is serving its users well.

## Availability and requirements

 is open for both academic and commercial use. The site was built with open source tools and libraries.

## Abbreviations

HTTP: Hyper-Text Transfer Protocol; JMX: Java Management Extensions; JVM: Java Virtual Machine; RDF: Resource Description Framework; REST: REpresentational State Transfer; RSS: Really Simple Syndication Format; TBD: To Be Done; TLD: Top-Level Domain; URI: Uniform Resource Locator; W3C: World Wide Web Consortium; XHTML: eXtensible HTML.

## Authors' contributions

EJ carried out most of the design and development work and drafted the manuscript. IP and SD participated in the development. EG, NR, AB, MJM, PM and BS coordinated design, requirements and specifications. EG, IP and NR helped to draft and critically revised the manuscript. All authors read and approved the final manuscript.
